# Conjunctival Autograft for Bilateral Tarsal Keratinization in a Case of Chronic Vernal Keratoconjunctivitis

**DOI:** 10.7759/cureus.23089

**Published:** 2022-03-12

**Authors:** Anahita Kate, Neha Jain, Saumya Jakati, Sayan Basu

**Affiliations:** 1 Cornea & Anterior Segment, L V Prasad Eye Institute, Vijayawada, IND; 2 Cornea & Anterior Segment, L V Prasad Eye Institute, Hyderabad, IND; 3 Ophthalmic Pathology Laboratory, L V Prasad Eye Institute, Hyderabad, IND; 4 Cornea, L V Prasad Eye Institute, Hyderabad, IND

**Keywords:** allergic conjunctivitis, lid margin, tarsal plate, keratinization, conjunctival autograft, vernal keratoconjunctivitis

## Abstract

This report describes the clinical features and management in a case of vernal keratoconjunctivitis (VKC) with bilateral tarsal conjunctival keratinization. A 32-year-old male presented with VKC since childhood that had exacerbated in the eight years prior to presentation. Examination revealed partial limbal stem cell deficiency in both eyes, with keratinization of the superior tarsal conjunctiva. The corresponding areas of the cornea exhibited punctate keratopathy in both eyes. To address this, the patient underwent excision of the conjunctival keratinization in both eyes. The resultant bare areas were covered with conjunctival autografts (CAGs). Postoperatively, the grafts were well apposed, and there was no recurrence of keratinization observed during the period of follow-up of four years. Resolution of corneal epitheliopathy was also noted. Although keratinization can occur in eyes with VKC, it is usually limited to the bulbar conjunctival areas. This is the first report of tarsal conjunctival keratinization in such cases. Milder cases may be observed or managed with scleral contact lenses. In more severe forms, there is associated corneal epitheliopathy, which may progress to corneal vascularization and scarring. Surgical excision of the lesion is recommended in these eyes. Following excision, several options exist to cover the bare area, which include a CAG, an amniotic membrane, or an oral mucous membrane. Of these, a CAG is an autologous tissue that can be harvested with a simple surgical technique and yields stable long-term results. Thus, tarsal conjunctival keratinization is a rare complication of chronic VKC. Excision of the lesion followed by a CAG is a viable approach for treatment, which reestablishes and maintains a stable ocular surface.

## Introduction

Vernal keratoconjunctivitis (VKC) is a chronic allergic inflammatory disorder that is associated with seasonal exacerbations and can cause serious visual impairment [1]. The disease process can predominantly involve the bulbar and/or the palpebral conjunctiva. In eyes with a chronic form of the disease, the resultant sequelae are also determined by this differential affliction. Giant papillae causing superior corneal epitheliopathy or shield ulcers are features of chronic palpebral VKC, while limbal stem cell deficiency (LSCD) and its associated epithelial healing issues ensue from the chronic limbal variant of VKC [1,2]. Symblepharon, sub-conjunctival fibrosis, and conjunctival keratinization can also develop in eyes with limbus involving VKC [2]. Furthermore, keratinization can be seen in VKC presenting with limbal pseudoepitheliomatous hyperplasia [3]. However, localized tarsal conjunctival keratinization in VKC has not been described before, and therefore this case aims to report the clinical features and management of bilateral tarsal conjunctival keratinization in eyes with VKC.

## Case presentation

**Figure 1:**
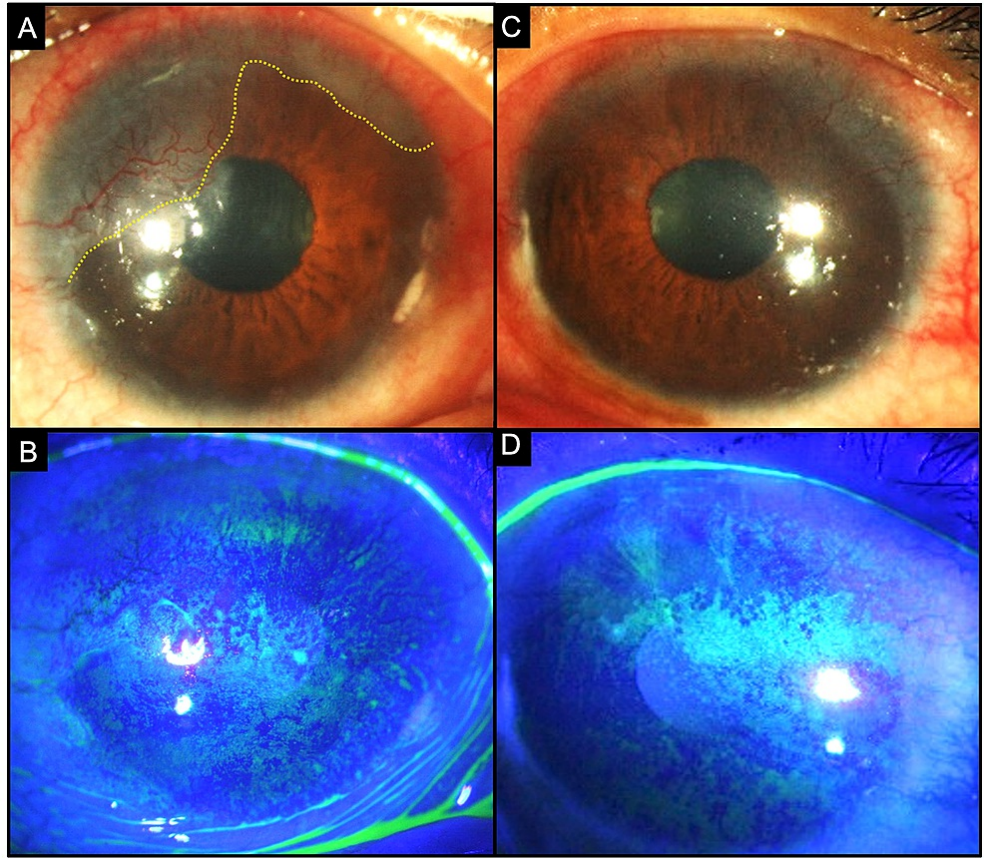
Collage of images depicting the clinical picture at presentation (A, B) Slit-lamp images of the right eye without and with fluorescein stain showing a congested conjunctiva with a pannus from 7 to 2 o’clock, which has extended over the corneal surface in the area in contact with the keratinization (yellow dotted line). Fluorescein-stained images depict denser superficial punctate keratitis superiorly. (C, D) Slit-lamp images of the left eye without and with fluorescein stain illustrating a pannus from 12 to 4 o’clock and coalescent staining of the corneal epithelium.

**Figure 2:**
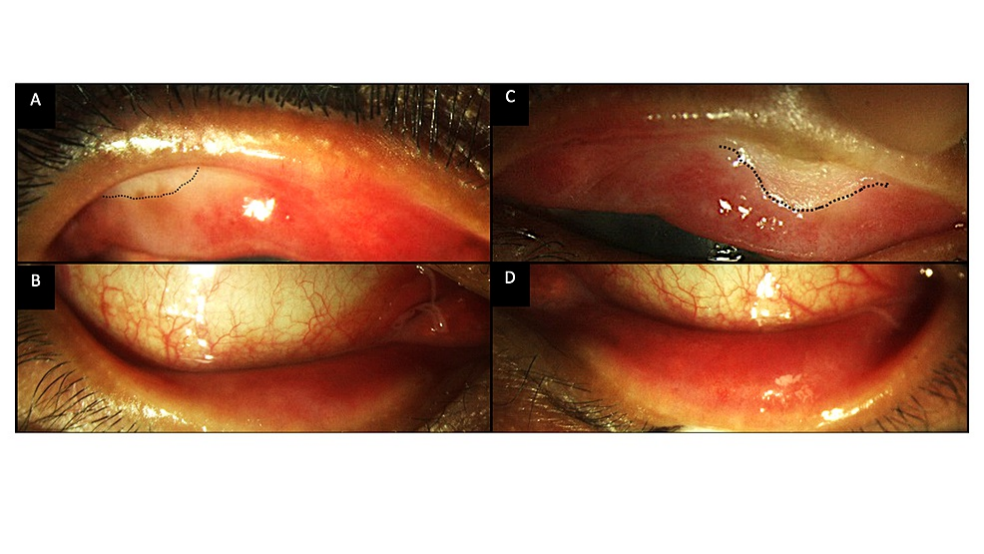
Clinical photographs of the everted upper and lower lids of both eyes (A, B) Images from the right eye depicting keratinization of the upper tarsal conjunctiva (black dotted line). The lower lid is uninvolved. (C, D) A similar picture is present in the left eye with keratinization of the upper lid (black dotted line).

A 32-year-old male presented with recurrent episodes of redness and itching since childhood, which had exacerbated in the eight years prior to presentation. He also had gradual painless decrease of vision in both eyes during the same period. There was no history of any systemic allergic disorder. At presentation, the best corrected visual acuity in both eyes was 20/40. On slit-lamp examination, both eyes showed conjunctival congestion with a fibrovascular pannus that extended from 7 to 2 o’clock in the right eye and from 12 to 4 o’clock in the left eye (Figure 1). There were no discernible palisades of Vogt in the remaining quadrants. On eversion of the upper lids, there was a presence of keratinization abutting the posterior lid margin in both eyes (Figure 2). The lower lids were uninvolved as were the lid margins of all four lids. The ocular surface was wet with a normal Schirmer’s value in both eyes. Staining of the corneal epithelium revealed diffuse punctate epithelial erosions that were denser in the superior part of the cornea in contact with the area of tarsal conjunctival keratinization. The rest of the anterior segment was within normal limits with an intraocular pressure of 14 mmHg and 12mmHg in the right and left eye, respectively. The examination of the posterior segment revealed no abnormalities. Based on these clinical findings, he was diagnosed to have VKC with LCSD in both eyes. The patient was started on topical corticosteroids (fluorometholone 0.1%) and lubricants (carboxymethylcellulose 0.5%). In order to address the keratinization, an excision of the lesion with a conjunctival autograft was planned.

**Figure 3:**
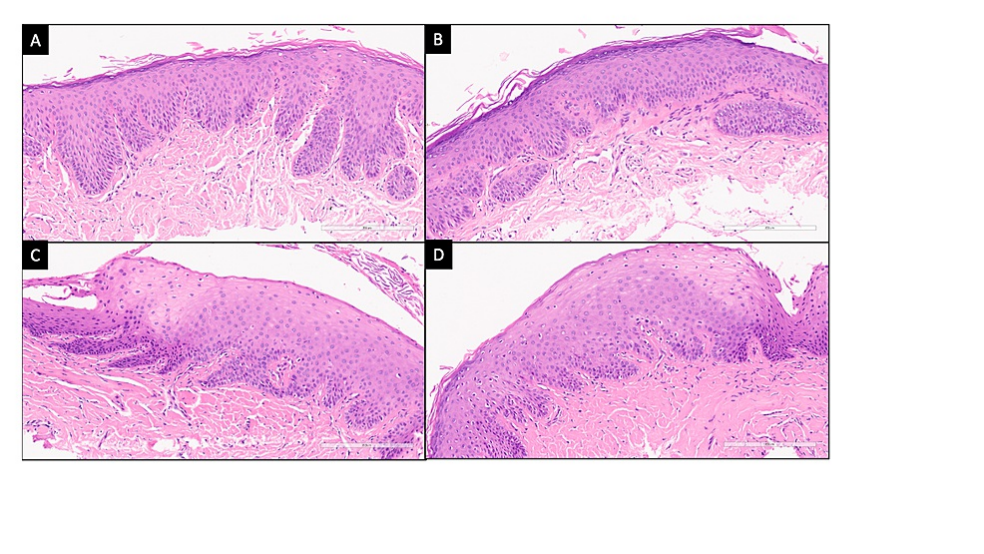
Photomicrographs from the excised conjunctival tissues from right eye (A, B) and left eye (C, D) show squamous metaplasia in the form of epithelial hyperplasia with downgrowth, loss of goblet cells, and surface keratinization. Subepithelial substantia propria shows dense fibrosis and dilated blood vessels (Hematoxylin and eosin; 20x original magnification).

The surgical procedure was performed under local anesthesia. In the right eye, a stay suture was passed with 6-0 silk in upper and lower lids to obtain adequate surgical exposure. The area of keratinization was separated from the underlying tissue by blunt dissection and excised. The sample was sent for histopathological examination. The bare area in the upper tarsal conjunctiva was measured to be 7x3mm. A small bleb was created in the inferior bulbar conjunctiva with Ringer's lactate solution and the conjunctiva was dissected from the underlying Tenon’s layer. A graft was harvested measuring the same size as the defect and secured with fibrin glue over the bare area (Tisseel Kit, Baxter AG, Vienna, Austria). The same procedure was repeated in the upper lid of the left eye. Histopathological examination of excised sample revealed marked epithelial hyperplasia with downgrowth, keratin deposits, and squamous metaplasia associated with sub-epithelial fibrosis in both eyes (Figure 3).

**Figure 4:**
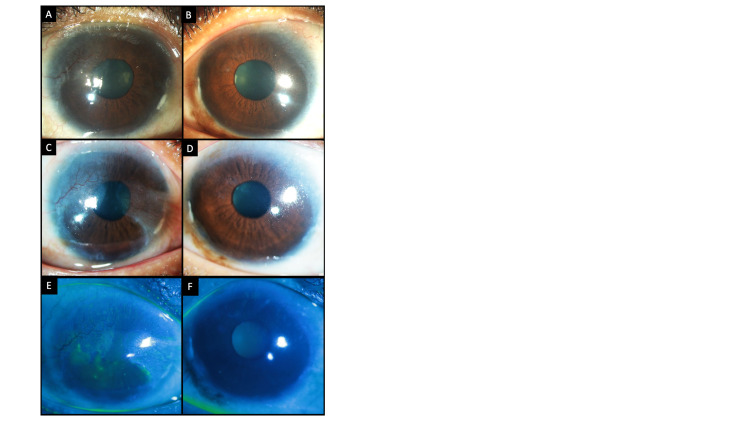
(A, B) Slit-lamp images of the right and left eyes, respectively, showing a stable ocular surface at the sixth postoperative week. (C-F) Images depicting the clinical picture four years after the initial presentation. (C) In the right eye, the temporal pannus is stable; however, progression of the nasal pannus is seen encroaching into the visual axis. (D) Left eye is well epithelialized with a quiet ocular surface. (E, F) Fluorescein-stained images of both eyes showing resolution of the punctate epitheliopathy of the superior corneal epithelium.

**Figure 5:**
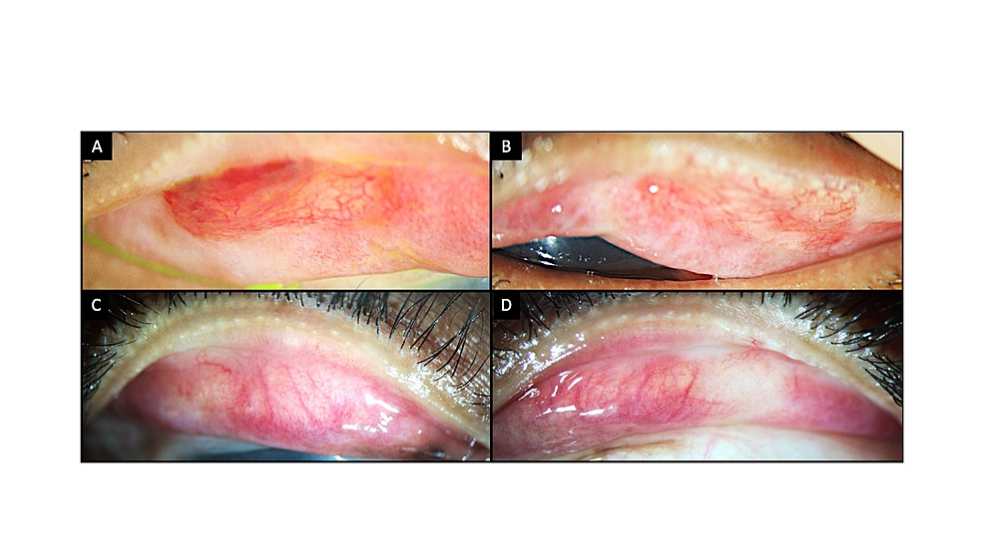
(A, B) Images of the everted right and left upper lids captured at the sixth week postoperatively showing a well-apposed conjunctival autograft. (C, D) Images captured four years after the initial presentation. No recurrence of keratinization is observed in the right and left upper lids respectively. The conjunctival grafts are in situ.

On the first postoperative day, the conjunctival grafts were in situ with an inferior conjunctival defect in both eyes. The patient was started on oral prednisolone 20mg once a day along with a topical antibiotic-steroid (chloramphenicol, polymyxin B, and dexamethasone) ointment at night, corticosteroids (prednisolone acetate 1%, 6 times/day), and lubricating agents (carboxymethylcellulose sodium 1%). The topical steroids were tapered weekly for six weeks. At the sixth week postoperative visit, there was no recurrence of the tarsal conjunctival keratinization, and the inferior conjunctiva was well epithelialized (Figures 4, 5).

The patient was prescribed oral prednisolone 5mg per day for three months along with topical antibiotic-steroid (chloramphenicol, polymyxin B, and dexamethasone) ointment at night, and lubricating agents (carboxymethylcellulose sodium 1%). A trial of Prosthetic Replacement of the Ocular Surface Ecosystem (PROSE) scleral lenses was given with which the visual acuity in the right and left eyes was 20/30 and 20/25, respectively.

The patient continued to follow up at regular intervals of six months for the next one year and had annual visits subsequently. There were two acute episodes of VKC: one at nine months and the other two years after the surgery. Hence, the patient was restarted on oral prednisolone 5mg per day. Topical immunosuppressants in the form of cyclosporine and tacrolimus were sequentially added with each episode. The patient was asymptomatic for two years with no further exacerbations of the disease on this regimen of immunosuppression (Figure 4). No systemic side effects were observed with the use of the oral steroids. Four years following the surgery, his visual acuity with the PROSE lenses was 20/25 in both eyes. The pannus in the right eye had progressed in the 2-4‘o clock area and was encroaching into the visual axis. No progression of the pannus was noted in the left eye. There was no recurrence of keratinization, and a stable ocular surface was observed at the last follow-up. Oral prednisolone (5mg/day) and topical cyclosporine 0.05% (two times/day) with tacrolimus eye ointment 0.03% (once at nighttime) were continued.

## Discussion

Keratinization of the ocular surface is usually seen in conditions wherein there is chronic stress to the surface epithelium. This could be secondary to inflammatory processes as in cases of Stevens-Johnson syndrome (SJS) or due to disorders affecting the epithelial turnover as seen in vitamin A deficiency. Rarely chronic irritation from topical medications can also cause keratinization of the ocular surface [4]. Keratinization is a rare complication of VKC and, when present, is usually seen on the bulbar conjunctiva [3]. On a histopathological level, this process manifests as metaplasia of the stratified non-keratinized columnar epithelium to keratinized squamous epithelium [5]. The exact causative factors that induce this change are unclear; however, several mechanisms have been postulated. These have been studied in the context of the more common causes of keratinization such as SJS and vitamin A deficiency. The inflammatory mediators recruited as a result of the chronic inflammation can activate the metaplasia of the non-keratinized epithelium [5]. The altered microbiome of the ocular surface in these eyes further contributes to this pathogenesis via enzymatic degradation of fatty acids [5]. A combination of these factors can be probable triggers of keratinization in VKC, though the presence of microbial dysbiosis in eyes with VKC is yet to be established [5-7].

Keratinization of the tarsal conjunctiva in VKC as seen in the present case has not been previously reported. Management of the same is based on its location and severity. If the keratinized areas are not in contact with the cornea, they can be observed with the use of lubricating agents to improve patient comfort. However, when the abnormal tarsal conjunctiva comes in contact with the cornea, it can abrade the corresponding corneal epithelium with every blink [8]. Repeated episodes of micro-abrasions can result in epithelial defects, neovascularization, and scarring, and thus is an indication for the treatment of the keratinization [8]. In the current case, superior corneal epitheliopathy was noted in both eyes corresponding to the area of keratinized conjunctiva. These repeated insults were particularly undesirable due to the presence of LSCD and its associated compromised epithelial healing. Thus, a prompt intervention was carried out.

Mild cases of keratinization can be managed with scleral contact lenses, which will protect the corneal epithelium and improve visual acuity by addressing the irregular astigmatism. In cases with significant keratopathy requiring surgical intervention, excision of the lesion in toto with an autologous conjunctival graft is a viable option. Although lid margin keratinization is traditionally treated with mucous membrane grafting, this is typically done in eyes with SJS or ocular cicatricial pemphigoid (OCP) where there is lack of healthy donor conjunctiva [9]. This is in contrast with eyes with VKC where a CAG can be easily harvested from the same eye or the fellow eye. Additionally, the surgical procedure with a CAG is simpler and the replaced tissue is homologous with better anatomical and functional outcomes. The use of a CAG has been extensively described for the management of pterygia, LSCD, etc., and has been established as an efficacious and safe procedure [10,11]. It has also been used in VKC following excision of giant papillae with good long-term outcomes [12]. As an autologous tissue is used, there is no risk of rejection or need for systemic immunosuppression. An amniotic membrane can also be used to cover the area of the defect, but this may result in the formation of a symblepharon and is dependent on the support of an eye bank. Thus, CAG is a simple and feasible surgical option in such cases with good clinical outcomes in the long term.

## Conclusions

Our case describes a rare case of isolated tarsal conjunctival keratinization in VKC, which was managed successfully with a CAG. Early intervention is recommended in eyes with secondary epitheliopathy to prevent formation of corneal scarring and vascularization. Conjunctival autograft is a viable surgical alternative to mucous membrane grafting in cases of localized tarsal conjunctival keratinization not associated with cicatrizing conjunctivitis. In the current case, this surgical procedure had a stable long-term outcome with no evidence of recurrence.
